# Assessing meaningful work among Hungarian employees: testing psychometric properties of work and meaning inventory in employee subgroups

**DOI:** 10.1186/s40359-022-00749-0

**Published:** 2022-03-07

**Authors:** Georgina Csordás, Balázs Matuszka, Viola Sallay, Tamás Martos

**Affiliations:** 1 Doctoral School, Semmelweis University, Budapest, Hungary; 2 Institute of Psychology, Eszterházy Károly Catholic University, Eger, Hungary; 3 Institute of Psychology, Pázmány Péter Catholic University, Budapest, Hungary; 4 Institute of Psychology, University of Szeged, Szeged, Hungary

**Keywords:** Meaningful work, WAMI, Confirmatory factor analysis, Psychometry

## Abstract

**Background:**

The construct of meaningful work (MW) has become the subject of various studies. Workers who experience MW have higher career and organizational commitment, report fewer days absent, and are characterized by a higher level of well-being. The aim of this study is to test a measure of MW, the Work and Meaning Inventory by Steger et al. This measure was created on theoretical background, and it constructs MW from three dimensions: psychological meaning, meaning-making, and greater good motivation.

**Methods:**

The analysis was conducted in a Hungarian sample (N = 2,498), using confirmatory factor analysis (CFA), and multiple-group CFA.

**Results:**

The three-dimensional model of the WAMI was confirmed in the analysis. In our study, the measure proved to be reliable, even in the test–retest analysis. Moreover, the discriminant and convergent validity of the WAMI was tested, with various relevant constructs: the presence and the search for life meaning, life satisfaction, and job satisfaction. Also a multiple-group CFA was conducted with the three-factor model, confirming measurement invariance regarding sex and working position.

**Conclusions:**

In line with the original version of the WAMI, the three-dimensional model was confirmed, with good psychometric properties in the Hungarian working context.

## Background

Nowadays, organizations and workplaces have started to evolve their perspectives on work. Reflecting on the uncertainty of the world (workers cannot rely on their workplaces as they did in the twentieth century), practitioners of vocational psychology developed new theories, emphasizing the significance of the perceived meaning of work. Organizations started to support these studies and practices due to the fact that meaningful work (MW, but also has been referred to as meaning in work [1]) has benefits both individually and organizationally. It has been found in several studies, that people who experience MW report fewer days absent, have higher career commitment, have fewer withdrawal intentions, and have better organizational commitment. MW also positively correlated with various well-being variables: workers who see their job as meaningful report higher satisfaction with their work and life overall and experience a higher sense of meaning in life. In addition, MW correlated negatively with depression and hostility [2–4]. In a study conducted with Chinese workers, the intrinsic aspirations predicted meaning in life through MW [5]. Moreover, in a recent study, MW predicted quality of life (life satisfaction and flourishing) through courage among Italian workers [6].

Even though MW became such a significant construct, there is little consensus about the meaning of MW. As Steger argues, MW has no founding figure like the concept of the meaning of life as proposed by Frankl [7]. According to a conceptual analysis presented by Both-Nwabuwe, Dijkstra, and Beersma [8], there are fourteen existing different definitions of MW. They propose that MW refers to the subjective experience of significance which comes from the fit between the worker and the work. However, different theories often approach MW as a multidimensional construct. For instance, Pratt, Pradies, and Lepisto [9] define MW through three orientations: craftmanship orientation, which refers to doing work for intrinsic motivational reasons, serving orientation, which means the value of work that comes from helping others, and kinship orientation, as the work creates bonds between colleagues. According to this approach, any of these orientations can provide the sense of MW.

In this paper, we define MW, according to Steger et al. [2] as MW refers to the overall perceived meaning people experience regarding their work. They distinguish meaning in work (MW) from the meaning of work. While MW answers the question “how meaningful is your work ?”, the meaning of work rather answers the “what makes your work meaningful?” [10]. In addition, they argue that despite the fact that scholars use the word “meaning” and “meaningful” interchangeably, one should refer to MW as the positive manifestations of meaningful work [7]. Moreover, the authors lay out that the existing models of MW blend the causes with the sources and the experience of MW [2]. For instance, in the job characteristic model developed by Hackman & Oldham [11] MW is a mediator between the job characteristics and the significance of the tasks. In the theoretical model of Rosso, Dekas, and Wrzesniewski [12], MW is attained by focusing on the self and others.

Steger et al. [2] created a model, which in contrast to previous models, attempts to define the experimental dimensions of MW. In their model, MW consists of three dimensions: positive meaning (PM) of work since the subjective experience of MW includes the perception that one’s work is meaningful and matters [12]. The second facet is meaning-making (MM) through work that characterizes how MW can theoretically contribute to one’s meaning in life. This captures the self-directed action in MW. The final dimension is greater good (GG) motivations, which is an other-directed action: one’s longing for making a positive impact in others’ lives.

### The work and meaning inventory

#### Measurements of meaningful work

There were studies regarding the operationalization of MW; for instance, Hackman and Oldham [11] have introduced a scale: however, it did not prove to be adequate [2]. Since then, the majority of MW scales were constructed by modifying the Hackman and Oldham’s measure. However, these scales were not developed on a clear theoretical framework, therefore not defining MW in a satisfactory way [2]. Hence there was a need for a reliable measure. Arnoux-Nicolas et al. [13] developed a questionnaire (Meaning of Work Inventory—MOW) based on three theoretical components (including the theoretical approach of Steger et al.), and validated it in French workers. MOW measures MW in four dimensions with fifteen items: the importance of work, understanding work, the direction of work, and the purpose of work.

The most widely used measure of MW is the Work and Meaning Inventory (WAMI) developed by Steger and colleagues [2]. Corresponding to their model of MW, the WAMI measures MW on three subscales: psychological meaningfulness, meaning-making, and greater good motivation. These dimensions were confirmed during their analysis, and the measure proved to be reliable and valid.

#### Language adaptations and psychometric properties of the work and meaning inventory

To this day, the WAMI was adapted to several languages with a diverse factor structure (see Table 1 below). The Turkish version was adapted to a sample of teachers from various educational institutes [14]. Reliability and validity were good in their sample, and the three-factor structure found by the original authors was replicated. The Italian version, which was conducted in a sample of workers from Tuscany, also found a three-factor structure and proved to be reliable and valid [15]. The factor structure of the latter was confirmed in a subsequent study although a model with the three-factor under a higher-order factor had better fit indices [16]. Adapting the WAMI to Polish language [17], researchers suggested a two-dimension model of MW in WAMI-PL: meaning in the self and meaning in world perspectives. However, the adaptation to a Brazilian sample of professionals [18] found a unifactorial model. The Romanian version was used in a model also as a single factor and proved to be reliable [19]. The latter findings suggest that the WAMI (and MW) have a different factor structure in samples from different cultures. These inconsistent cultural findings strengthen the necessity of a study in Hungarian culture.

**Table 1:** Summary of the psychometric properties of different language adaptations of the WAMI

Language	N	Number of factors	RMSEA	CFI	Cronbach's alpha value
Turkish [14]	352	3	0.087	0.98	0.64–0.86
Italian [15]	344	3	0.070	0.92	0.74–0.82
Polish [17]	393	2	0.051	0.98	0.88, 0.92
Brasilian [18]	667	1	0.080	0.99	0.96
Romanian [19]	235	1	0.060	0.92	0.9

#### Findings with the WAMI

The first study conducted with the WAMI was by Steger et al. [2], accompanied by the first analysis of the measure. A significant positive correlation was found with the concept of calling and calling-orientation and a negative one with calling-seeking. The authors also found a clear differentiation from experiencing or seeking calling. As for well-being variables, they found a positive connection with the presence of meaning and life satisfaction and a negative correlation with hostility and depression. The connection between the WAMI and various work-related variables was also examined, where a positive correlation was found with career and organizational commitment, organizational citizenship behaviors, job satisfaction and the presence of intrinsic motivation, and negative connection with days reported absent, withdrawal intentions from the organization as well from occupation. The results were similar in association with the subscales, except that counter to the other subscales, MW had no significant correlation with calling seeking and career orientation. Leonardo et al. [18] found similar results according to intrinsic motivation (positive correlation with large effect), and work commitment (also strong positive connection), and besides this, a positive connection with occupational self-efficacy. A study conducted in a Chinese sample supports this finding, namely participants who valued intrinsic aspirations experienced more MW and meaning in life in general [5]. Akin et al. [14] found a positive association with medium power between total the WAMI scores and job crafting, which was defined as self-initiated changes made by workers in favor of aligning their work with their motives, preferences and personal goals [20]. In a recent study in Turkey [21], researchers explored variables which help reduce the feeling of loneliness among nurses. They found that among communication frequency, social interactions, and the trust in leaders, MW played an important role as well. In a Polish sample, Puchalska-Kamińska et al. [17] also found congruent results with the previous studies regarding meaning in life, well-being indicators, such as organizational commitment, work engagement, and positive work behaviors like extra-role and in-role behaviors as well as job crafting. In a research study conducted with middle manager workers in India, MW proved to have a mediating effect between job design and work engagement. According to this study, an effective job design amplifies MW, which leads to stronger work engagement [22]. A study conducted in Romania also found a positive connection between MW and work engagement and a negative correlation with intent to leave [19]. In a study in Belgium, researchers measured MW with only two items of the PM scale in the WAMI (even so the reliability of the scale proved to be good with a Cronbach alpha value = 0.78). They found strong positive correlations between MW and work-related resources (autonomy, strengths use, needs-supply fit and future-orientedness of the job). Moreover, the study indicates that psychological needs are direct predictors of MW [23].

### The Hungarian context and the need for a reliable measure for MW

Psychological measures can be affected by the culture it was developed in. If we adopt a questionnaire into Hungarian, we must consider the Hungarian context. Regarding job involvement, it seems to be less important to Hungarian workers than in other countries, according to a cross-cultural study in 2000 [24]. Furthermore, it was found that all situational variables in connection with one’s economic position predict motivation, and salaries predict responsibility. Besides this, no evidence was found to support a motivational after-effect of communism. In 2003, a study [25] found that autonomy and charisma play lesser roles in the expectations regarding leadership and management in Hungary, in contrast to German or other Central-European leadership environments. They argue that Hungary shows similarity with European Latin countries like Portugal, Spain, or Italy. Others suggest that these tendencies lead to the conclusion that more controlling methods of leadership are favored in Hungary [26]. Counter to the international tendencies, there are few studies in Hungary regarding MW. One of them [27] found a negative correlation between depression and MW, where the latter construct was measured by the Work organization and job contents subscale of the second version of The Copenhagen Psychosocial Questionnaire [28]. The subscale contains three questions regarding MW, for instance: *“Is your work meaningful?”*. In consideration of the proven significance of MW, there is a need for more research in the field, and for that, a reliable measure as well.

### Aims of the study

The purpose of this research is to present the Hungarian version of the WAMI and examine its psychometric properties. Furthermore, we test the measure in employee subgroups in order to get a wider picture according to the nature of MW in the Hungarian working population. The measure was adapted to various languages with various cultural backgrounds, and there is an inconsistency regarding the factor structure of the measure. We aim to adapt the questionnaire to the Hungarian context, providing a reliable and valid scale; measuring the construct of MW.

## Methods

### Sample

Altogether 2,498 respondents completed the survey. The mean age was 40.25 years (*SD* = 11.583), ranging from 19 to 77 years. In terms of sex, 47.1% (*N* = 1177) were male, and 52.9% (*N* = 1321) were female. More than half of the sample had college or university-level education, and nearly one-quarter of the sample were high school graduates. The majority of the sample worked as an employee (68.78%), 17.49% as a leader, and nearly 9% as a freelancer. In our sample, 13.73% of the participants worked in leading positions; in sum, 56.77% were white-collar workers, 7.81% were employed as skilled workers, and nearly 5.3% were unskilled laborers. Almost 1% of the sample worked in agriculture, 2.96% were freelancers and entrepreneurs and 3% were students (for more information see Table 2).

**Table 2:** Highest level of education, job position, and occupation in the sample

Highest level of education			Position			Occupation		
Value	Frequency	Percent	Value	Frequency	Percent	Value	Frequency	Percent
Elementary level education or lower	10	0.4	Leader	437	17.49	In leading position	343	13.73
Vocational school	108	4.3				Intellectual worker	518	20.74
High school graduate	552	22.1	Employee	1718	68.78	Other white-collar worker	900	36.03
						Skilled worker	195	7.81
						Unskilled worker and laborer	132	5.29
Higher-level vocational training	291	11.65	Freelancer	224	8.97	Agricultural laborer	19	0.76
						Freelancer, entrepreneurs (not agricultural)	74	2.96
						Student	75	3.00
College or university level education (or higher)	1439	57.6						
Other	–	–	Other	119	4.76	Other	240	9.60
Missing	98	3.92	Missing	–	–	Missing	2	0.08
Total	2498	100	Total	2498	100	Total	2498	100

### Data collection

Participants were Hungarian workers recruited via snowball method, with the help of university students from the University of Szeged and Pázmány Péter Catholic University. Data were collected through an online survey from 2014 to 2018. Prior to data collection, approval of the United Ethical Review Committee for Research in Psychology (EPKEB 2014/28) was obtained. The inclusion criteria were being at least 18 years old and working in a job. At the beginning of the assessment process, participants were informed in writing about the general topic of the research and that the participation was anonymous and voluntary and could be terminated at any time. Afterward, participants gave their informed consent in writing to participate in the study. At the beginning of the survey, sociodemographic variables were included, e.g., age, sex and the highest level of education.

### Measures

#### Meaning in life questionnaire (MLQ)

The questionnaire measures one’s perceived meaning of life. It was developed by Steger et al. [29]. Since then, it has been widely used in various studies. The MLQ consists of 10 items (one of them is reversed) in two subscales with five items for each dimension: the presence of meaning, and search for meaning. Respondents used a 7-point scale. Items are rated from 1 (absolutely untrue) to 7 (absolutely true). The scale appears to be reliable and has good convergent and discriminant validity. The measure was adapted to Hungarian by Konkolÿ Thege and Martos [30].

The two-factor model suggested by Steger et al. [2] was tested with conducting CFA, with the following results: χ^2^[34] = 953.48 (χ^2^/df = 28.04), RMSEA = 0.106, SRMR = 0.106, CFI = 0.93, TLI = 0.912.^1^ As for reliability, we used Cronbach’s alpha coefficient. The value of the Presence factor was 0.911, and the value of the Search factor was 0.837, indicating good reliability.

#### Satisfaction with life scale (SWLS)

The SWLS was developed to measure global life satisfaction by Diener et al. [31]. It is commonly used in positive psychological research. The scale has only five items (none of them are reversed), which measures in a 7-point scale, rated from 1 (strongly disagree) to 7 (strongly agree). The Hungarian adaptation was made by Martos et al. [32]. They found that the Hungarian version has good internal consistency and validity.

The measurement model of the scale was tested in our study. The results of the CFA were as follows: χ^2^[5] = 111.81 (χ^2^/df = 22.36), RMSEA = 0.094, SRMR = 0.020, CFI = 0.985, TLI = 0.969. The RMSEA indicated a mediocre fit, while the others indicated a good fit. Cronbach’s alpha value was 0.892, indicating excellent internal consistency.

#### Satisfaction with work scale (SWWS)

This scale was developed from the items of the SWLS, but instead of the satisfaction with life, it measures the satisfaction with one’s work. It also consists of five items without reversed items and uses a 7-point Likert scale (1 = strongly disagree, 7 = strongly agree). The reliability of the SWWS was found good in a previous research [26].

CFA was conducted to measure the factorial validity of the SWWS since no previous research performed it. The indices were: χ^2^[5] = 153.27 (χ^2^/df = 30.654), RMSEA = 0.111, SRMR = 0.032, CFI = 0.974, TLI = 0.949. Except for the RMSEA, the other indices suggest a good fitting model. These findings were similar to the results of SWLS. Cronbach’s alpha coefficient was 0.866.

#### The work and meaning inventory (WAMI)

The measure was developed by Steger et al. [2]. The original version of the questionnaire measures the Meaning of Work (MW) in three subscales: Positive Meaning (PM), Meaning Making (MM), and Greater Good motivations (GG). The WAMI has ten items (one item is reversed) and measures in a 5-point Likert-type scale. The main purpose of this study is to adapt the questionnaire to the Hungarian language and measure its psychometric properties. The first step was translating the items by independent professionals who are experts in this area. Second, the final version was made by extended discussions between the translators and the principal investigator after the re-translation process.

### Data analysis

The preliminary and bivariate analyses were conducted in IBM SPSS Version 25. For confirmatory factor analyses and multigroup analyses, we used JASP statistical software Version 0.14.1.0. [33]. Researchers use measurement invariance to investigate group differences of latent variables. While cross-cultural studies are trending, group comparisons within a single culture are of little interest, though some argue that these are required in order to interpret differences reliably [34]. Multiple-group confirmatory factor analysis (MG-CFA) is the most common method to investigate if a measure is invariant across groups. The testing for measurement invariance is a series of model comparisons, where strict and stricter constraints are defined [35]. First, a baseline model, second, a weak-invariance (also known as metric) model, then a strong invariance (scalar) model [36]. Following the guidelines of Chen [37], we used the comparative fit index (CFI) as a criterion to define invariance, with the cutpoint of change below 0.01 in CFI, as it seems to be the most used and empirically best-supported criterion. Additionally, we also used the likelihood ratio test for invariance testing. However, this test is sensitive to sample size and may lead to false-positive results in large sample sizes. To prevent the undue rejection of invariance and to quantify the magnitude of the difference between the models, we calculated an effect size value (*w*), which is based on Cohen’s effect size measure. The interpretation of *w* is similar to Cohen’s d, with *w* = 0.1 indicating small, *w* = 0.3 medium, and *w* = 0.5 indicating large effect [38].

## Results

### Confirmatory factor analysis

We tested the three-factor model reported by Steger et al. [2] and compared its fit with the unifactorial structure that was indicated by previous studies [18, 19]. The fit indices were better in the three-dimensional model. The chi-square tests were significant: however, the test is sensitive to sample size, and rejects models with large sample sizes [38, 39]. SRMR was acceptable in both models, while the RMSEA indicated a poor fit in both models. The incremental fit indices (CFI and TLI) indicated mediocre, and a good fit in both cases (above 0.95 is a good fit [40]. A detailed comparison can be seen in Table 3.

**Table 3:** Summary of fit indices for confirmatory factor analysis

Fit indices	χ^2^	df	χ^2^/df	RMSEA	SRMR	CFI	TLI
1-factor model	2011.94						
	*p* < .001	35	57.48	0.151	0.062	0.863	0.823
3-factor model	1516.54						
	*p* < .001	32	47.39	0.136	0.052	0.897	0.855
3-factor model with covariances	632.87						
	*p* < .001	29	21.82	0.091	0.037	0.958	0.935

We also ran the analysis including a second-order factor to the model, with the three-factor (PM, MM, and GG) load on a second-order latent variable. The fit indices were the same as in the three-factor model, indicating that the total score of the WAMI can be used to measure MW. This result also suggests that the covariances indicated by the modification indices are caused by the wording of the items.

**Fig. 1:**
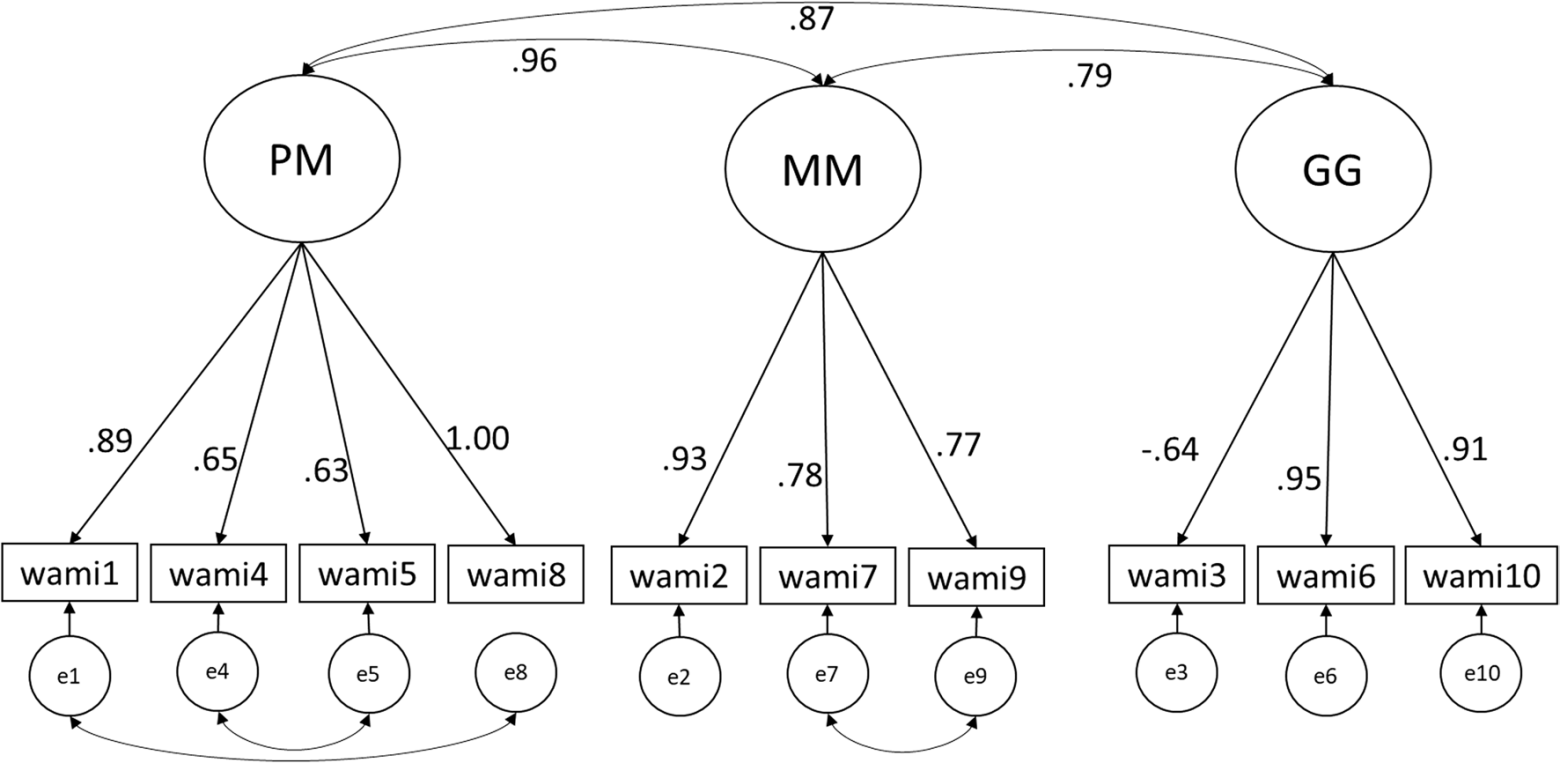
Model plot of the three-factor model

To achieve a better fit, we included covariances between certain items (wami1 and wami8, wami4 and wami5, wami7 and wami9). The decision was based on the modification indices and on the overlapping meaning of the items which may have caused shared variance that could account for the shared covariances [41]. For instance, wami7 (“My work helps me better understand myself.”) and wami9 (“My work helps me make sense of the world around me.”) are items of the MM factor, in which the contribution of work to the meaning of life is articulated. However, in the Hungarian version of the questionnaire, the expression “understanding” is also used for expressing “make sense of” thus this identical phrasing could be a source of the shared variance. The model plot of the final model can be seen at Fig. 1.

#### Multiple-group confirmatory factor analysis

We ran the MG-CFA analysis to examine the possible differences between males and females. According to the CFI values, the item loadings and intercepts are similar in the groups (see Table 4). The *w* values indicate that the significant results of the chi-square test are due to the large sample size.

**Table 4:** Summary of the multiple-group factor analysis between sex and job positions

Model	χ2	df	p	w	CFI (ΔCFI)
*Results of the multiple-group factor analysis between sex*					
M1 configural	672.223	58	< 0.001		0.95743
M2 metric	689.516	65	< 0.001	0.031	0.95671 (0.00072)
M3 scalar	705.577	72	< 0.001	0.030	0.95609 (0.00062)
*Results of the multiple-group factor analysis between job positions*					
M1 configural	727.6	87	< 0.001		0.95341
M2 metric	748.934	101	< 0.001	0.024	0.95288 (0.0012)
M3 scalar	780.559	115	< 0.001	0.030	0.9516 (0.00128)

We also ran MG-CFA with the three main job position subgroups: leader, employee, and freelancer. According to the CFI, there is an equivalent variability across the groups (see Table 4). This implies that the item loadings and intercepts, are similar in the groups [42]. The low effect-size values (*w*) indicate that the significant chi-square values are due to the large sample size. The factor loadings range from 0.630 (item 3) to 0.993 (item 8).

### Reliability

To investigate the internal consistency reliability, Cronbach’s alpha coefficient was used. Cronbach’s alpha values were as follows: PM = 0.862, MM = 0.814, GG = 0.745, MW(total score) = 0.843, indicating good reliability.

Furthermore, we measured the test–retest reliability of the scale to see if the WAMI can produce consistent results with the same group of people tested at different points in time (on average two weeks passed between the measurements). For that, we used Pearson’s correlation coefficients. We conducted the analysis with 41 participants. All the pairs of the test and retest items correlated significantly. Item level correlations ranged from 0.335 to 0.724 (for items 3 and 6, respectively). The correlation between the total score test and retest values was 0.747. As for the subscales, we found significant positive correlations (PM = 0.745, MM = 0.660, GG = 0.480).

### Divergent and convergent construct validity

To examine the divergent and convergent validity of the measure, we used measures of constructs that are associated with MW. We found a significant positive correlation with medium effect between the WAMI total scores and satisfaction with life (SWLS) scores, and a strong positive one with satisfaction with work (SWWS) scores. As for the MLQ, there was a positive correlation with a medium effect between the WAMI and the Presence of the meaning subscale but we did not find a significant correlation with the Search for meaning subscale. The correlations were similar for the WAMI subscales, except for the Search for meaning subscale, which correlated significantly with the subscales: we found a strong negative connection with PM and GG, and a positive one with MM. The results are detailed in Table 5.

**Table 5:** Correlations between the WAMI Subscales, SWLS, SWWS, and MLQ Subscales

	WAMI	PM	MM	GG
SWLS	0.383***	0.386***	0.346***	0.279***
SWWS	0.571***	0.613***	0.490***	0.398***
MLQ-presence	0.426***	0.436***	0.338***	0.349***
MLQ-search	− 0.038	− 0.083***	0.042*	− 0.052*

WAMI—Work and meaning inventory, PM—work and meaning inventory positive meaning subscale, MM- work and meaning inventory meaning making subscale, GG—work and meaning inventory greater good subscale, SWLS—satisfaction with life scale, SWWS—satisfaction with work scale, Presence—meaning in life questionnaire presence subscale, search—meaning in life questionnaire search subscale

*p < .05, ***p < .001

### Descriptive statistics and subgroup differences

We tested for sex differences using independent samples t-test. We found a significant difference in the case of the following variables: the WAMI total score, MM, GG, SWLS, MLQ total and MLQ Presence. However, in all cases, the effect size values indicated very small effects (the largest Cohen’s d value was -0.121; in that case, the difference in means was only 1.01 points between the groups). For more details see Table 6.

**Table 6:** Descriptive statistics and sex differences for the scales in the study

	Full sample		Male		Female		Male–Female comparison	
	M	SD	M	SD	M	SD	t	Cohen’s d
PM	15.42	3.53	15.31	3.48	15.53	3.58	− 1.48	− 0.06
MM	10.50	2.93	10.24	2.87	10.73	2.97	− 4.03***	− 0.17
GG	10.98	2.86	10.82	2.73	11.12	2.96	− 2.470*	− 0.103
WAMI total	36.90	8.27	36.37	8.00	37.38	8.50	− 2.919**	− 0.121
SWWS total	21.74	6.87	21.76	6.71	21.73	7.02	0.10	0.001
SWLS total	23.38	6.59	22.91	6.66	23.80	6.51	− 3.29**	− 0.14
MLQ presence	26.43	6.79	25.91	6.79	26.90	6.75	− 3.51***	− 0.15
MLQ search	21.86	7.43	21.59	7.26	22.12	7.57	− 1.71	− 0.07
MLQ total	48.23	9.08	44.65	8.39	45.74	8.25	− 3.18**	− 0.13

WAMI—Work and meaning inventory, PM—work and meaning inventory positive meaning subscale, MM- work and meaning inventory meaning making subscale, GG—work and meaning inventory greater good subscale, SWWS—satisfaction with work scale, SWLS—satisfaction with life scale, MLQ—meaning in life questionnaire, Presence—meaning in life questionnaire presence subscale, Search—meaning in life questionnaire search subscale

*p < 0.05, **p < 0.01, **p < 0.001

The descriptive statistics of the WAMI scores in the main position subgroups are shown in Table 7.

**Table 7:** WAMI scores in the main job position subgroups

		PM	MM	GG	WAMI total
Leader	M	16.37	11.20	11.52	39.09
	SD	3.07	2.58	2.69	7.37
Employee	M	15.04	10.22	10.77	36.03
	SD	3.60	2.98	2.88	8.38
Freelancer	M	16.56	11.35	11.50	39.40
	SD	3.42	2.86	2.95	8.15

WAMI—Work and meaning inventory, PM—Work and meaning inventory positive meaning subscale, MM–work and meaning inventory meaning making subscale, GG—work and meaning inventory greater good subscale

## Discussion

In this study, we examined the psychometric properties of the Hungarian version of the WAMI. We tested both the three- and one-factor models via confirmatory factor analysis, which indicated a better fit in the three-dimensional model. Supplementary analyses were made with the three-dimensional model, supported by the confirmatory analysis. Cronbach’s alpha value confirmed the reliability of the scale. The construct validity of the measure was proven; we found positive connections with satisfaction with life and work, and with the presence of meaning. These findings are similar to the original model proposed by Steger et al. [2], they are in line with the results of the Turkish [14] and the Italian [15, 16] samples. However, in the Polish sample, the WAMI seems to have a two-dimensional structure [17], while in other cultures, a unidimensional one (e.g. [18]). In our study, we also conducted a multiple-group confirmatory factor analysis to determine if there was any invariance in the sample. According to our findings, the measure has equivalent variability in terms of sex and job positions. We can conclude that the WAMI has the same psychometric characteristics in different subgroups of Hungarian employees.

### Limitations of the study

First, similarly to many other psychological studies, one limitation was the recruitment process. The data assessment procedure was a non-probability sampling method with online snowball recruitment of potential respondents. This may have resulted in the exclusion of marginal layers of the population and an overrepresentation of workers with college or university level education. In our study, we obtained conflicting results during the factor analyses. Also, the cross-sectional sample has limitations since it represents the sample at a given time. The fit of the MLQ-H was lower than optimal, although estimates of internal consistency were comparable to previous studies [30, 43, 44]. Furthermore, we investigated the connection between MW and other constructs via correlation methods. Thus, we cannot conclude any causality.

## Conclusions

Meaning of work is now in the focus of several studies, benefiting both organizations and employees. The specific significance of the study is that it provides data on work experiences from an Eastern-Central European country, thus expanding the cross-cultural comparability of the previous findings. In this study, we presented the Hungarian version of the Work and Meaning Inventory, a much-needed measure that proved to be reliable and valid. Complementary to the original version of the WAMI, we found the three-dimensional structure of the measure most sufficient. Further research should focus on the relations of MW and other related constructs, with a more representative sample.

The WAMI adds a new color to the available psychometric tools to the Hungarian professional community to assess the level of MW across diverse groups of employees in a feasible way in order to develop targeted interventions at an individual, group- or organizational level. Furthermore, with its established reliability and validity properties the WAMI can serve as an important benchmark tool to demonstrate efficacy in all sorts of activities in connection with workplace physical- and mental health: organizational development procedures, workplace-based health-promotion programs and in individual counseling processes.

## Data Availability

The datasets used and/or analyzed during the current study are available from the corresponding author on reasonable request.

## Appendix

### Appendix 1 Abbreviations, English and Hungarian Version of WAMI items

**Table:**

Abbreviation	Original item	Hungarian item
wami1	I have found a meaningful career	Egy értelmes hivatást találtam
wami2	I view my work as contributing to my personal growth	Úgy érzem, hogy a munkám hozzájárul a személyes fejlődésemhez
wami3	My work really makes no difference to the world.*	A munkám egyáltalán nincs hatással a világra.*
wami4	I understand how my work contributes to my life’s meaning	Tisztában vagyok azzal, hogy a munkám miképp járul hozzá az életem értelmessé tételéhez
wami5	I have a good sense of what makes my job meaningful	Jól tudom, hogy mi teszi értelmessé a munkámat
wami6	I know my work makes a positive difference in the world	Tudom, hogy a munkám pozitív változásokat eredményez a világban
wami7	My work helps me better understand myself	A munkám segít abban, hogy jobban megértsem önmagam
wami8	I have discovered work that has a satisfying purpose	Rátaláltam egy olyan munkára, amelynek értelmes célja van
wami9	My work helps me make sense of the world around me	A munkám segítségével jobban megértem a körülöttem lévő világot
wami10*	The work I do serves a greater purpose	A munka, amit végzek, egy nagyobb cél szolgálatában áll

*Reversed item.

## Abbreviations

GG: Greater good motivations
MG-CFA: Multiple-group confirmatory factor analysis
MLQ: Meaning in life questionnaire
MM: Meaning-making through work
MW: Meaningful work
PM: Positive meaning
SWLS: Satisfaction with life sale
SWWS: Satisfaction with work scale
WAMI: Work and meaning inventory

## Footnotes

1 According to the modification indices, mlq10 (*“I am searching for meaning in my life.”),* which is an item of the Search factor, may also negatively load on the Presence factor. If we allow the cross-loading, the absolute fit indices improve to an acceptable level: RMSEA = .082, SRMR = .076. However, this modification does not correspond to the original model.
